# Parathyroid gland volume and treatment resistance in patients with secondary hyperparathyroidism: a 4-year retrospective cohort study

**DOI:** 10.1093/ckj/sfae391

**Published:** 2025-01-10

**Authors:** Kazuhiko Kato, Akio Nakashima, Masamitsu Morishita, Ichiro Ohkido, Takashi Yokoo

**Affiliations:** Division of Nephrology and Hypertension, Department of Internal Medicine, Jikei University School of Medicine, Tokyo, Japan; Division of Molecular Epidemiology, Jikei University School of Medicine, Tokyo, Japan; Department of Nephrology, Morishita Memorial Hospital, Kanagawa, Japan; Division of Nephrology and Hypertension, Department of Internal Medicine, Jikei University School of Medicine, Tokyo, Japan; Department of Nephrology, Morishita Memorial Hospital, Kanagawa, Japan; Division of Nephrology and Hypertension, Department of Internal Medicine, Jikei University School of Medicine, Tokyo, Japan; Division of Nephrology and Hypertension, Department of Internal Medicine, Jikei University School of Medicine, Tokyo, Japan

**Keywords:** calcimimetics, parathyroid gland, secondary hyperparathyroidism, treatment resistance, ultrasonography

## Abstract

**Background:**

The role of parathyroid gland (PTG) ultrasonography in the management of secondary hyperparathyroidism after the introduction of calcimimetics remains unclear. Recent investigations have prompted renewed interest in the use of PTG ultrasonography for assessing treatment resistance to calcimimetics and determining the optimal timing for surgical intervention. This study aimed to explore the hypothesis that the PTG volume correlates with the calcimimetic dose.

**Methods:**

We retrospectively observed outpatients undergoing haemodialysis at baseline and a 4-year follow-up. PTG volume was measured using ultrasonography between January and December 2017 and January and December 2021. We examined the association between baseline PTG volume and calcimimetic doses after 4 years.

**Results:**

Of the 121 patients {median age 64 years [interquartile range (IQR) 54–72]}, 71 had PTG nodules on ultrasonography and the median total PTG volume was 34 mm^3^ (IQR 0–178). In the short dialysis vintage group, baseline parathyroid hormone levels tended to correlate with baseline calcimimetic doses; however, this trend was not observed in the extended dialysis vintage group. Baseline PTG volume correlated with the cinacalcet-equivalent calcimimetic dose (correlation coefficient 0.46; *P* < .001) after 4 years. The calcimimetic dose in the group with an estimated PTG volume >500 mm^3^ was ≈80 mg/day higher than that in the non-PTG nodule group after 4 years. In multivariate linear regression analysis, PTG volume >500 mm^3^ was associated with a high calcimimetic dose at 4 years in all analysis models.

**Conclusions:**

Assessing PTG volume using ultrasonography may help predict high calcimimetic doses.

## Graphical Abstract

**Graphical Abstract:**
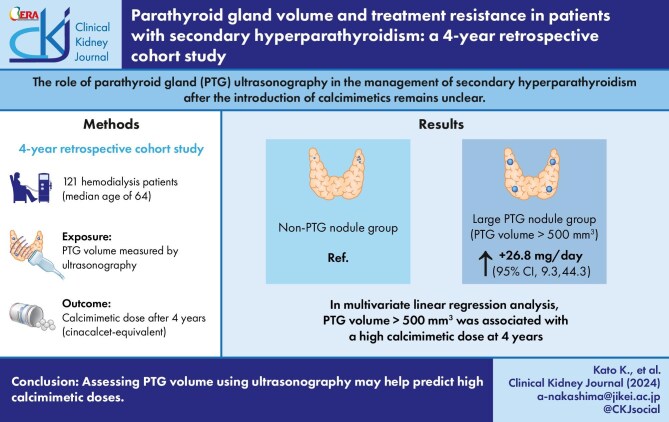

KEY LEARNING POINTS **What was known:**

- Since the introduction of calcimimetics, the role of parathyroid gland (PTG) ultrasonography in the management of secondary hyperparathyroidism (SHPT) has been unclear.
- Recent studies have prompted a renewed interest in the clinical utility of PTG ultrasonography for assessing treatment resistance to calcimimetics and determining the optimal timing for surgical intervention.

**This study adds:**

- PTG nodule size was associated with calcimimetic dose after 4 years.
- Using the degree of enlargement along with baseline parathyroid hormone levels provided a more accurate prediction of high calcimimetic doses in SHPT patients after 4 years.
- Our findings indicate the relevance of PTG ultrasonography in predicting calcimimetic doses.

**Potential impact:**

- This study suggests that the need for ultrasonography persists despite the paradigm shift introduced by calcimimetics.

## INTRODUCTION

Secondary hyperparathyroidism (SHPT), caused by impaired vitamin D activation and phosphate excretion associated with renal dysfunction, is central to the pathology of chronic kidney disease–mineral and bone disorder in patients undergoing haemodialysis (HD). This condition increases the risk of fractures and induces extensive organ damage via calcification [1].

Parathyroid gland (PTG) hyperplasia associated with SHPT evolves from the diffuse to nodular form with disease progression [2–4]. The quantitative evaluation of nodular hyperplasia through non-invasive ultrasonography facilitates the assessment of SHPT severity and estimation of parathyroid hormone (PTH) production [5]. Notably, several studies conducted in the 2000s to 2010s showed that gland size is a critical parameter in evaluating the efficacy of vitamin D or its analogues for SHPT [6–9].

The introduction of calcimimetics marked a significant shift in the treatment paradigm for SHPT, transitioning from reliance on vitamin D and parathyroidectomy (PTX) to calcimimetics. Initially, the relevance of measuring the PTG size decreased, given the reduced need for surgical intervention owing to these medical advancements [10, 11]. Nonetheless, recent research has highlighted the prognostic advantage of PTX over medical therapy alone [12–14], prompting a renewed interest in the clinical utility of PTG imaging to evaluate treatment resistance to calcimimetics and to determine the optimal timing for surgical intervention.

Several clinical studies have reported an association between PTG volume and calcimimetics [15–25]. Nevertheless, these findings are limited by small sample sizes, brief observational durations and a lack of studies incorporating newer agents such as evocalcet or etelcalcetide [6]. To address these limitations, we conducted a 4-year retrospective observational cohort study starting in 2017, as the baseline year, in patients on HD to explore the hypothesis that PTG volume is associated with the calcimimetic dose.

## MATERIALS AND METHODS

### Ethical considerations

All procedures involving human participants were approved by the institutional review board of the Jikei University School of Medicine (approval number 34-145[11296]) and conducted in accordance with the ethical standards of the 1964 Helsinki Declaration and its later amendments. Owing to the retrospective study design, the need for informed consent was waived by the institutional review board.

### Patient inclusion

We defined the inclusion criteria as patients who received HD at Morishita Memorial Hospital, Kanagawa, Japan, between 2017 and 2021, and the exclusion criteria as patients who had undergone parathyroidectomy, those without parathyroid ultrasonography and those for whom clinical data were difficult to obtain.

### Clinical parameters

Data on demographic characteristics, comorbidities and current medications were collected based on the nearest time point of data derived from ultrasonography. Blood tests were performed at the start of the week prior to HD. Laboratory parameters including creatinine (mg/dl), albumin (g/dl), calcium (mg/dl), phosphate (mg/dl), alkaline phosphatase (U/l; measured using the Japan Society of Clinical Chemistry method) and intact PTH (pg/ml) were measured. Owing to the high variability in the PTH measurement, the average of two blood tests conducted closest to the 2017 PTG ultrasonography was used as the baseline level and the average values at 36, 42 and 48 months were used as the outcomes for PTH levels after 4 years in each analysis. Based on previous studies, evocalcet and etelcalcetide doses were converted to cinacalcet-equivalent calcimimetic doses [26, 27]. High-resolution ultrasonography was used to assess the PTG volume at baseline and 4 years later (Aplio i600 TUS-AI600; Canon Medical Systems, Otawara, Japan). Measurements were performed by four well-trained clinical laboratory technicians using the following protocol: detectable PTGs were measured in the anterior–posterior (*a*), transverse (*b*) and longitudinal (*c*) dimensions. We calculated the estimated PTG volume using the following formula: estimated PTG volume = π/6 × *a* × *b* × *c*.

### Statistical analyses

Data are presented as mean ± standard deviation or median and interquartile range (IQR). Patients were divided into three groups: those with no PTG identified by baseline ultrasonography (non-PTG nodule group), those with identified PTG and an estimated total PTG volume ≤500 mm^3^ (small PTG nodule group) and those with an estimated PTG volume >500 mm^3^ (large PTG nodule group), which is the cut-off value in the guidelines in Japan [28]. Differences in background characteristics among the groups were assessed using the χ^2^ test for binary variables and analysis of variance (ANOVA) and the Kruskal–Wallis test was used for continuous variables. Next, we visualized the changes in PTH levels using dot plots, displaying 99% of all patients, with the IQR indicated by a red line, and tested for differences using a repeated measures ANOVA employing the Greenhouse–Geisser correction. Additionally, we analysed PTG volume changes in subgroups of patients whose PTG volumes were measurable at both ultrasonography sessions. The groups were divided based on median age, sex, dialysis history, baseline PTH levels and calcimimetic doses. Subsequently, the associations of baseline PTG volume with PTH levels and cinacalcet-equivalent calcimimetic dose 4 years later were assessed by determining correlation coefficients and drawing scatter plots and regression lines. Additionally, the correlations of baseline PTG volumes with mean calcium, phosphate, calcium × phosphate product and PTH levels in the observation period were assessed using correlation coefficients, scatter plots and regression lines. Subsequently, we used receiver operating characteristics (ROC) curves to show predictive accuracy, with PTH >240 pg/ml (target upper limit of the Japanese guideline [28]) and a high dose of calcimimetics (cinacalcet-equivalent dose >50 mg/day) at 4 years as outcomes. Baseline PTH levels alone (red) and with the addition of the categorical variable PTG volume (blue) were illustrated and areas under the curve (AUC) were determined for each variable. Subsequently, multivariate linear regression analysis was performed to examine the independent association between baseline PTG ultrasound findings (categorical variables) and calcimimetic doses or PTH levels after 4 years. The disjunctive cause criterion was used as the covariate selection method and variables that were the causes of exposure, outcomes or both, according to previous studies, were selected as confounders [29]. In model 1, sex, age, body mass index and dialysis vintage were added to the unadjusted model. In model 2, calcium, phosphate, albumin and intact PTH levels were added to the variables used in model 1. In model 3, the baseline administration of calcium-based phosphate binders, non-calcium-based phosphate binders, active vitamin D analogues and baseline calcimimetic dose were added to the variables used in model 2. None of the variables exhibited multicollinearity. Finally, scatter plots were drawn to examine the interplay between dialysis vintage, PTH and calcimimetic dose. The plots showed the relationships between baseline PTH levels and baseline calcimimetic dose and between baseline PTG volume, PTH levels and the calcimimetic dose after 4 years, with patients categorized into two groups based on the median dialysis vintage. All tests were two-sided, with *P* < .05 indicating statistical significance. A complete case analysis was performed when data were missing. Because this study was exploratory, no adjustments for multiplicity were planned. All statistical analyses were performed using Stata version 15.1 (StataCorp, College Station, TX, USA).

## RESULTS

### Patient characteristics

The study included 160 patients. After excluding 6 patients who underwent PTX, 1 patient who had not undergone ultrasonography and 32 patients for whom clinical data were difficult to obtain, 121 patients remained for analysis (Supplementary Fig. S1). The median age of the 121 patients was 64 years (IQR 54–72), with a median dialysis vintage of 88 months (IQR 31–162) (Table 1). Seventy-one patients with PTG nodules were identified using ultrasonography, and the median total PTG volume was 34 mm^3^ (IQR 0–178). Elevated calcium levels were observed in the group with PTG nodules (*P* = .016) at a median level of 8.5 mg/dl (IQR 7.9–9). The median phosphate level was 5.1 mg/dl (IQR 4.1–6.1), with no significant differences noted between groups. The median PTH level was 159 pg/ml (IQR 110–263). PTH levels tended to rise as the PTG volume increased (*P* = .191). The PTG groups had higher albumin and creatinine levels (*P* = .003 and *P* < .001, respectively). Phosphate binders were administered orally to 74.4% of the patients, with significantly more administration in the PTG nodule groups (*P* < .001). Additionally, calcimimetics were internally administered to 47.9% of the patients, and the number of patients on calcimimetics and doses were significantly higher in the PTG nodule groups (*P* = .004 and *P* = .003, respectively).

**Table 1:** Baseline patient characteristics stratified by PTG ultrasound findings (*N* = 121).

Characteristics	Overall	Non-PTG nodule group (*n* = 50)	Small PTG nodule group (estimated total PTG volume ≤500 mm^3^; *n* = 60)	Large PTG nodule group (estimated total PTG volume >500 mm^3^; *n* = 11)	*P*-value
Total PTG volume (mm^3^), median (IQR)	34 (0–178)	0 (0–0)	88 (43–258)	840 (675–969)	<.001
No. of PTG nodules, *n* (%)					<.001
0	50 (41.3)	50 (100)	0 (0)	0 (0)	
1	29 (24)	0 (0)	29 (48.3)	0 (0)	
2	22 (18.2)	0 (0)	19 (31.7)	3 (27.3)	
3	15 (12.4)	0 (0)	10 (16.7)	5 (45.5)	
4	5 (4.1)	0 (0)	2 (3.3)	3 (27.3)	
Age (years), median (IQR)	64 (54–72)	65 (53–74)	63.5 (55.5–69)	54 (44–73)	.407
Male, *n* (%)	82 (67.8)	34 (68)	41 (68.3)	7 (63.6)	.953
Body mass index (kg/m^2^), median (IQR)	21.9 (19.5–24.9)	21.3 (18.7–25.3)	22.1 (20.2–25.1)	23.4 (20.9–24.2)	.506
Smoking history, *n* (%)	66 (54.6)	25 (50)	40 (66.7)	1 (9.1)	.001
Dialysis vintage (months), median (IQR)	88 (31–162)	52 (15–89)	102 (49–170)	171 (126–245)	<.001
Diabetes mellitus, *n* (%)	52 (43)	25 (50)	24 (40)	3 (27.3)	.312
Laboratory test results					
Albumin (g/dl), mean ± SD	3.6 ± 0.4	3.5 ± 0.5	3.7 ± 0.3	3.8 ± 0.2	.003
Creatinine (mg/dl), median (IQR)	11.8 (9.8–14)	10.8 (8.3–12.7)	12.5 (10.4–14.5)	14.1 (12.5–14.6)	<.001
Calcium (mg/dl), median (IQR)	8.5 (7.9–9)	8.2 (7.8–8.6)	8.6 (8–9.2)	9 (8.3–9.3)	.016
Phosphate (mg/dl), median (IQR)	5.1 (4.1–6.1)	5.4 (4.3–6.4)	5.1 (4–5.8)	5.4 (4.7–6.2)	.541
ALP (IU/ml), median (IQR)	231 (188–317)	247.5 (199–327)	228.5 (186–320)	204 (168–275)	.263
Intact PTH (pg/ml), median (IQR)	159 (110–263)	157 (89–264)	159 (110.5–253.5)	225 (151–340)	.191
Treatment for CKD-MBD					
Calcium-based phosphate binders, *n* (%)	56 (46.3)	22 (44)	30 (50)	4 (36.4)	.646
Phosphate binders (non-Ca), *n* (%)	90 (74.4)	28 (56)	51 (85)	11 (100)	<.001
Active vitamin D analogues (oral + intravenous), *n* (%)	81 (66.9)	33 (66)	40 (66.7)	8 (72.7)	.92
Calcimimetics, *n* (%)^a^	58 (47.9)	18 (36)	30 (50)	10 (90.9)	.004
Dose of cinacalcet equivalent (mg/day), median (IQR)	0 (0–25)	0 (0–25)	6.25 (0–25)	25 (25–75)	.003

ALP: alkaline phosphatase; Ca, calcium.

^a^Only one patient in the non-PTG nodule group was on etelcalcetide, all others were on cinacalcet.

### Changes in PTH levels and PTG volume

**Figure 1:**
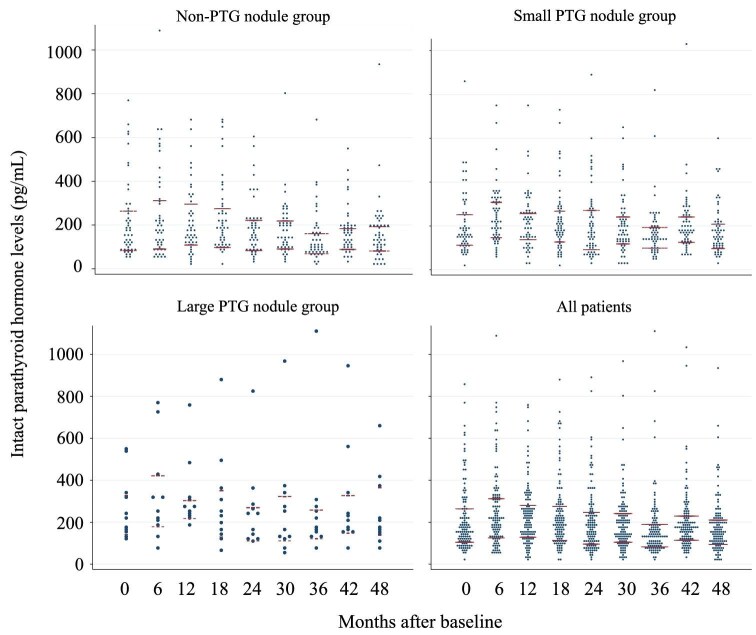
PTH level trends divided by the baseline PTG volume.

Figure 1 shows the changes in PTH levels over 4 years, with the PTG nodule groups (small PTG nodule and large PTG nodule groups) showing significantly higher PTH levels than the non-PTG nodule group (*P* = .036). Additionally, of the 50 patients in the non-PTG nodule group, 14 had PTG nodules on the 4-year ultrasonography. The estimated PTG volume in the 14 patients was 63 mm^3^ (IQR 33–259). The PTH level of these patients at 4 years was 162 pg/ml (IQR 112–225), which was not significantly different from the baseline levels and was not noticeably higher in the non-PTG nodule group. Conversely, in four patients in the PTG nodule group, the 4-year ultrasonography failed to identify PTG nodules. Subgroup analysis showed a trend toward parathyroid regression in patients with a longer history of dialysis, higher baseline PTH levels and higher calcimimetic doses (Supplementary Table S1).

### Correlation of the PTH level and calcimimetics dose with PTG volume

**Figure 2:**
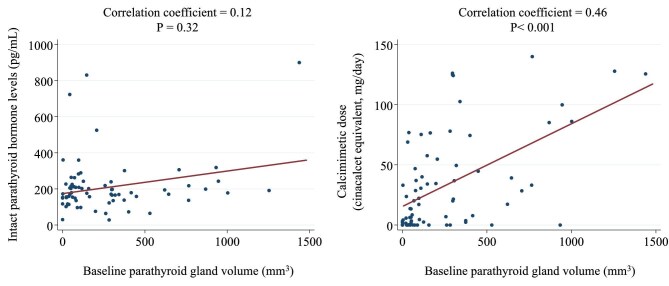
Correlation of PTH levels and calcimimetic dose after 4 years with baseline PTG volume.

Figure 2 shows the correlations between the PTH level, calcimimetic dose after 4 years and baseline PTG volume. The correlation coefficient between the PTH levels and PTG volume was 0.12 (*P* = .32) and that between the calcimimetic dose and PTG volume was 0.46 (*P* < .001).

### Correlation of the mean calcium, phosphate, calcium × phosphate product and PTH levels with PTG volume

Baseline PTG volume weakly correlated with mean phosphate levels (correlation coefficient 0.24; Supplementary Fig. S2).

### ROC curves to predict the severity of secondary hyperparathyroidism

**Figure 3:**
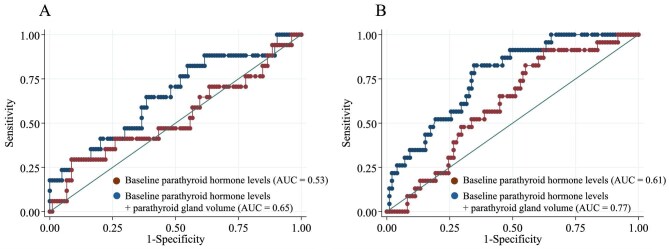
ROC curves predicting **(A)** PTH levels >240 pg/ml and **(B)** cinacalcet-equivalent calcimimetic dose >50 mg/day after 4 years.

Figure 3 shows the ROC curves predicting PTH levels >240 pg/ml and the cinacalcet-equivalent calcimimetic dose >50 mg/day after 4 years. Compared with predictions based on baseline PTH levels alone, adding PTG volumes increased the sensitivity, specificity and AUC (Fig. 3A: from 0.53 to 0.65; Fig. 3B: from 0.61 to 0.77).

### Multivariate linear regression analysis

In the linear regression analysis of the association between baseline PTG volume and PTH levels at 4 years (Table 2), PTH levels were ≈40 pg/ml higher in the large PTG nodule group than in the non-PTG nodule group. PTG volume >500 mm^3^ was significantly associated with elevated PTH levels at 4 years, except in multivariate model 3 (*P* = .08). In the linear regression analysis of the association between baseline PTG volume and calcimimetic dose at 4 years (Table 3), the calcimimetic dose was ≈80 mg/day higher in the large PTG nodule group than in the non-PTG nodule group. PTG volume >500 mm^3^ was associated with a high calcimimetic dose at 4 years in all analysis models. In some analysis models, PTG volume <500 mm^3^ was also associated with high calcimimetic doses compared with those in the non-PTG nodule group.

**Table 2:** Univariate and multivariate linear regression analyses of the baseline PTG ultrasound findings (categorical variable) in relation to PTH levels after 4 years.

	Non-PTG nodule group (*n* = 50)	Small PTG nodule group (estimated total PTG volume ≤500 mm^3^; *n* = 60)			Large PTG nodule group (estimated total PTG volume >500 mm^3^; *n* = 11)		
Intact PTH levels	143 (104–175)	170 (134–215)			182 (163–293)		
(pg/ml), median (IQR)							
		β	95% CI	*P*-value	β	95% CI	*P*-value
Unadjusted	Reference	40.2	−8.3–88.6	.103	116.5	32.3–200.8	.007
Model 1	Reference	35.1	−14.6–84.9	.164	105.6	17.4–193.8	.019
Model 2	Reference	31.9	−20.4–84.3	.229	94.3	2.3–186.3	.045
Model 3	Reference	28	−27.6–83.5	.32	88.3	−10.7– 187.3	.08

CI: confidence interval.

Model 1: Sex, age, body mass index and dialysis vintage were added to the unadjusted model.

Model 2: Calcium, phosphate, albumin and intact PTH levels were added to the variables in model 1.

Model 3: The baseline administration of calcium-based phosphate binders, phosphate binders (non-calcium) and active vitamin D analogues and the baseline dose of calcimimetics were added to the variables in model 2.

**Table 3:** Univariate and multivariate linear regression analyses of the baseline PTG ultrasound findings (categorical variable) in relation to the calcimimetic dose after 4 years.

	Non-PTG nodule group (*n* = 50)	Small PTG nodule group (estimated total PTG volume ≤500 mm^3^; *n* = 60)			Large PTG nodule group (estimated total PTG volume >500 mm^3^; *n* = 11)		
Calcimimetic dose	0 (0–13.9)	12.5 (0–43.8)			81.4 (26.4–126.5)		
(mg/day), median (IQR)							
		β	95% CI	*P*-value	β	95% CI	*P*-value
Unadjusted	Reference	16.8	5.1–28.5	.005	59.6	39.4–79.9	<.001
Model 1	Reference	14	2.4–25.6	.019	51.5	30.8–72.1	<.001
Model 2	Reference	10.9	−0.9–22.7	.069	44.6	23.9–65.3	<.001
Model 3	Reference	7.7	−2.1–17.5	.123	26.8	9.3–44.3	.003

CI: confidence interval.

Model 1: Sex, age, body mass index and dialysis vintage were added to the unadjusted model.

Model 2: Calcium, phosphate, albumin and intact PTH levels were added to the variables in model 1.

Model 3: The baseline administration of calcium-based phosphate binders, phosphate binders (non-calcium) and active vitamin D analogues and the baseline dose of calcimimetics were added to the variables in model 2.

### Subgroup analysis by dialysis vintage

In the short dialysis vintage group, baseline PTH levels tended to correlate with calcimimetic doses; however, this trend was not observed in the extended dialysis vintage group (Supplementary Fig. S3). Additionally, the relationship between PTG volume and calcimimetic dose after 4 years was more prominent among patients with an extended dialysis vintage (Supplementary Fig. S4).

## DISCUSSION

The present study showed that the PTG nodule groups persistently maintained high PTH levels and that the nodule size was associated with the calcimimetic dose after 4 years. Moreover, incorporating the degree of enlargement with baseline PTH levels provided a more accurate prediction of calcimimetic doses in patients with SHPT at the 4-year time point. The utility of PTG ultrasonography remains a topic of debate in the modern era, especially with calcimimetics playing a central role in treating SHPT. This study underscores the necessity for ultrasonography despite the paradigm shift introduced by calcimimetics.

When comparing background factors, the baseline biochemical data revealed higher calcium levels in the PTG nodule group and a trend toward higher PTH levels, although the differences were not significant. This group also consumed more oral phosphate binders and calcimimetics, indicating a trend toward greater SHPT severity.

Figure 2 shows that the degree of PTG volume correlated with the calcimimetic dose at 4 years in patients with identifiable PTG nodules at baseline. In contrast, PTH levels were not associated with PTG volume. This could be because patients with higher PTH levels were appropriately treated with calcimimetics per the guidelines [28].

Figure 3 demonstrates that incorporating the PTG volume (as a categorical variable) with baseline PTH levels enhanced the accuracy of predicting the requirement for high calcimimetic doses as well as elevated PTH levels at 4 years. Multivariate linear regression analysis showed an independent association between baseline PTG volume, PTH levels and calcimimetic doses after 4 years (Tables 2 and 3). These findings suggest that measuring the PTG volume using ultrasonography could be valuable for predicting long-term calcimimetic doses and determining the optimal timing for surgical intervention.

The subgroup analysis showed a correlation between PTH levels and calcimimetic doses in patients with a short dialysis vintage, consistent with the findings of previous studies [30]. At the same time, this relationship was absent in those with extended dialysis vintage. This finding suggests that prolonged calcimimetic treatment facilitates the effective suppression of hyperparathyroidism. Nonetheless, even in patients with prolonged dialysis vintage, significant parathyroid hyperplasia required higher calcimimetic doses, indicating that measuring parathyroid volume could be particularly valuable in this population.

PTG hyperplasia is linked to the progressive downregulation of calcium-sensing receptors (CaSRs) and vitamin D receptors [31–33]. Additionally, cinacalcet enhances CaSR expression. However, this effect was inhibited in patients with enlarged PTGs along with nodular hyperplasia, suggesting a physiological mechanism that confers resistance to calcimimetics [34].

Literature on the relationship between PTG nodules and cinacalcet treatment resistance is sparse, with most studies reporting small cohorts of 10–30 patients [15–17]. More extensive studies involving nearly 60 patients had short follow-up periods ranging from 7 months to 2 years [18, 19]. Thus, further investigations with longer durations were necessary to clarify the role of PTG nodules in resistance to SHPT treatment. This study addresses this gap by reporting a 4-year observational investigation of 121 patients, representing the largest cohort examined to date, to assess the association between PTG volumes and calcimimetic doses.

In the case of refractory SHPT, PTX has been documented to enhance life expectancy [12–14] by improving PTH management. Nonetheless, many eligible patients do not undergo surgery and continue medical management [35, 36], likely because of the absence of a definitive threshold for surgical intervention. When SHPT is difficult to manage medically, Japanese guidelines recommend surgical intervention if the estimated PTG volume is >500 mm^3^ or 1 cm in length [28]. Conversely, the Kidney Disease: Improving Global Outcomes (KDIGO) guidelines do not address parathyroid volume as an indication for PTX [1]. However, utilizing minimally invasive and readily accessible ultrasound [37] to detect PTG nodule size, which puts patients at risk of SHPT treatment resistance, and determining the optimal surgical intervention timing may positively impact life expectancy. This study offers additional evidence supporting this approach.

Although PTG nodule size is linked to calcimimetic doses, previous reports have suggested that calcimimetics exert a regressive effect on the PTG. Cinacalcet administration in rats decreased both the number and volume of PTG cells [38]. Furthermore, human observational studies reported decreased PTG volumes following cinacalcet treatment [20, 21]. Aside from the cell volume decrease due to the diminished demand for PTH synthesis [21], it has been proposed that cinacalcet may induce PTG cell apoptosis [39]; however, the precise mechanisms remain unclear. The observed trend toward PTG regression in patients with high baseline PTH levels receiving calcimimetics in the present study may support this hypothesis. However, a randomized prospective trial is needed to further investigate this hypothesis.

The interpretation of this study may need to consider the specific characteristics of dialysis management in Japan. The Japanese guidelines [28] recommend maintaining PTH levels between 60 and 240 pg/ml, which is lower than the KDIGO guidelines [1] that suggest keeping PTH levels within 2–9 times the assay's upper limit. Indeed, the average PTH levels in Japan are lower than in other regions, including Europe and the USA [40], with many patients in the present study having levels <300 pg/ml. Furthermore, because of the limited number of cases (*n* = 121), only two patients had an average PTH level >500 pg/ml. Therefore, extrapolating these results to patients from other countries and different ethnic backgrounds may be challenging.

This study has some limitations. First, causal relationships could not be determined because of the observational design. Second, ultrasonography was performed by four technicians trained under a consistent protocol to minimize bias. Nonetheless, the potential for differences in test sensitivity between operators cannot be excluded. Third, almost all patients with calcimimetics at baseline were being treated with cinacalcet; after 4 years, most had switched to evocalcet or etelcalcetide, with the calcimimetic doses calculated using the correction formula described in the Methods section, based on prior studies. However, the reliability of this formula must be improved. Fourth, because measuring PTG volumes annually is challenging, volume changes were assessed at two time points: baseline and after 4 years. Fifth, since we retrospectively analysed clinical data using ultrasonography to measure PTG volumes, we could not explore more accurate methods, such as nuclear medicine imaging. Sixth, since calcium levels influence PTH secretion, conducting the analysis in subgroups stratified by calcium levels would have been ideal. Additionally, we considered testing the mediating effects of PTH on the relationship between calcium levels and PTG volume. Furthermore, to solely examine the effects on PTG volume, it would have been helpful to analyse only patients who did not receive calcimimetics before study enrolment. However, we could not perform these analyses due to the limited number of cases. Finally, this study could not assess outcomes, such as deaths or fractures. Therefore, future prospective studies should be conducted to further elucidate the relationship between PTG volume and resistance of SHPT to treatment as well as the underlying mechanisms.

In conclusion, assessing PTG volume using ultrasonography may aid in predicting future calcimimetic doses.

## Supplementary Material

sfae391_Supplemental_Files

## Data Availability

The data underlying this article will be shared upon reasonable request to the corresponding author.
