# Supplementary material for: Parathyroid gland volume and treatment resistance in patients with secondary hyperparathyroidism: a 4-year retrospective cohort study
Source: Clin Kidney J. 2025 Jan 10;18(2):sfae391. doi: 10.1093/ckj/sfae391 (PMC11803308; doi:10.1093/ckj/sfae391)
Supplement: sfae391_Supplemental_Files [file sfae391_supplemental_files.zip › Supple figure legends.10.29.docx]

**Supplementary Figure Legends**

Supplementary Figure S1. Patient flowchart

Supplementary Figure S2. Correlation of chronic kidney disease-mineral and bone disorder parameters with baseline parathyroid gland volume

Supplementary Figure S3. Correlation of the calcimimetic dose with parathyroid hormone levels stratified by median dialysis vintage

Supplementary Figure S4. Correlation of the parathyroid hormone levels and calcimimetic dose after 4 years with baseline parathyroid gland volume stratified by median dialysis vintage
