# Supplementary material for: Parathyroid gland volume and treatment resistance in patients with secondary hyperparathyroidism: a 4-year retrospective cohort study
Source: Clin Kidney J. 2025 Jan 10;18(2):sfae391. doi: 10.1093/ckj/sfae391 (PMC11803308; doi:10.1093/ckj/sfae391)
Supplement: sfae391_Supplemental_Files [file sfae391_supplemental_files.zip › Supplemental Figure 1.pdf]

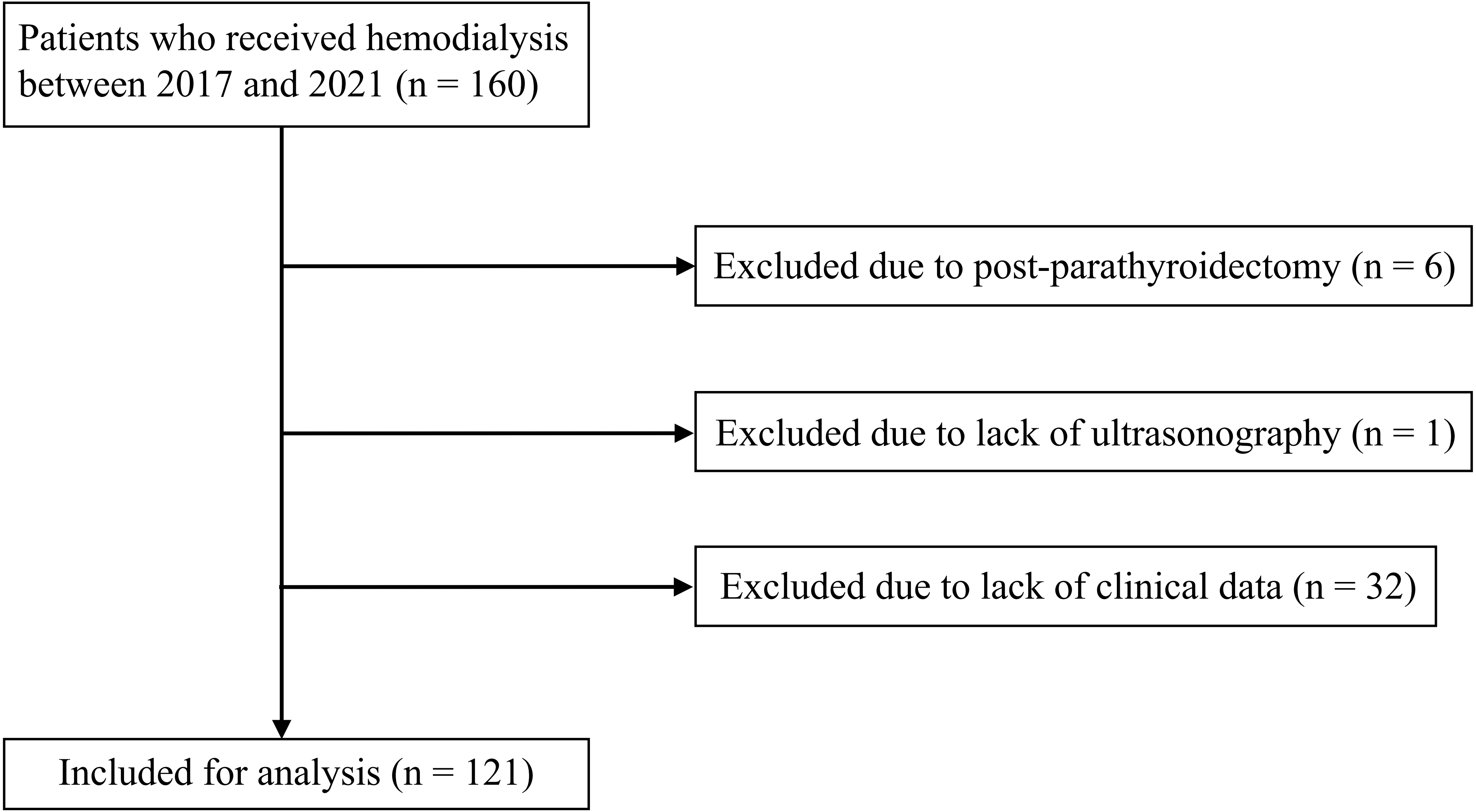
Patients who received hemodialysis between 2017 and 2021 (n = 160)
Excluded due to post-parathyroidectomy (n = 6)
Excluded due to lack of ultrasonography (n = 1)
Excluded due to lack of clinical data (n = 32)
Included for analysis (n = 121)
