# Supplementary material for: Parathyroid gland volume and treatment resistance in patients with secondary hyperparathyroidism: a 4-year retrospective cohort study
Source: Clin Kidney J. 2025 Jan 10;18(2):sfae391. doi: 10.1093/ckj/sfae391 (PMC11803308; doi:10.1093/ckj/sfae391)
Supplement: sfae391_Supplemental_Files [file sfae391_supplemental_files.zip › Supplemental Figure 2.pdf]

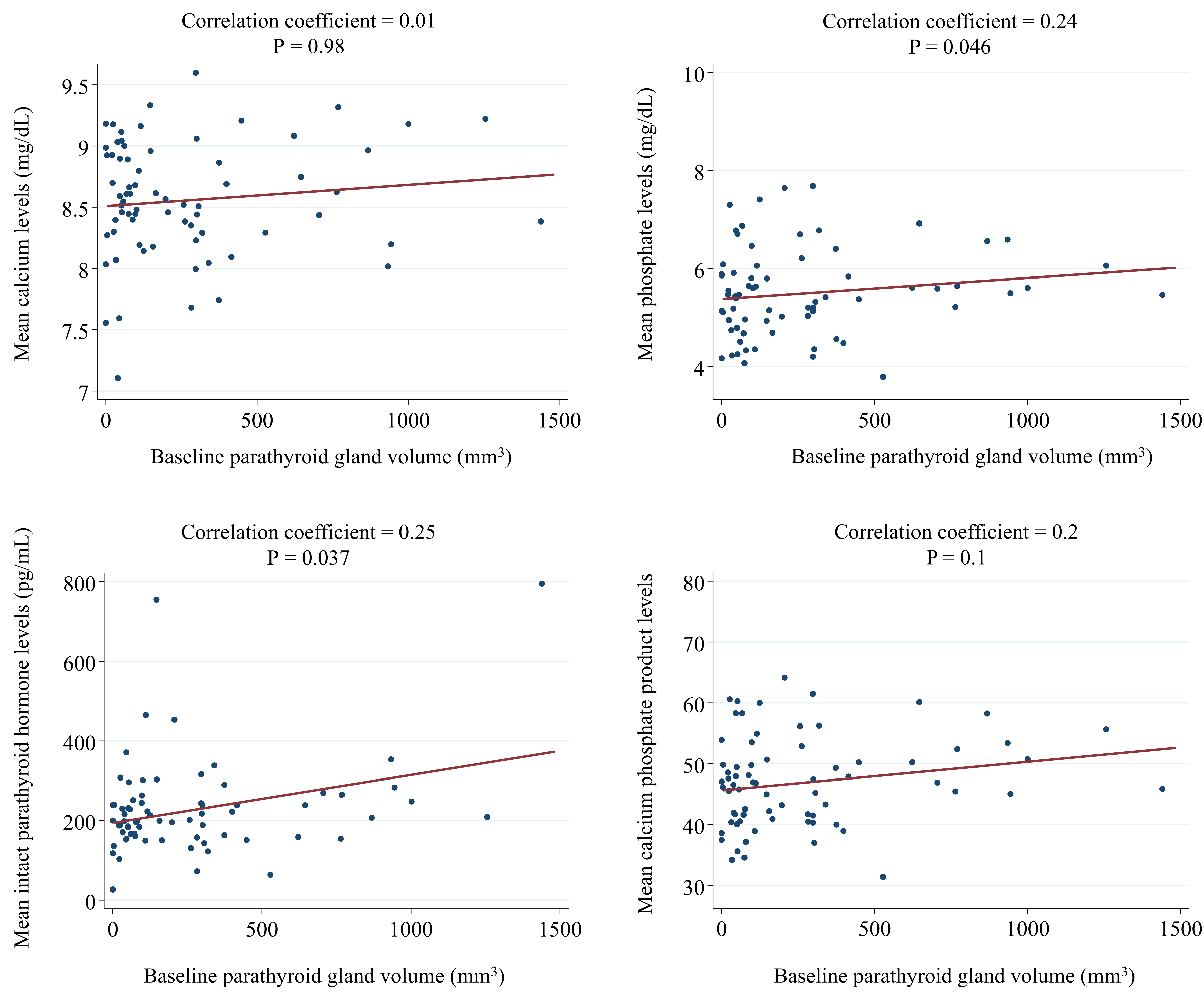

Correlation coefficient = 0.01
P = 0.98
Mean calcium levels (mg/dL)
Baseline parathyroid gland volume (mm^3)
Correlation coefficient = 0.24
P = 0.046
Mean phosphate levels (mg/dL)
Baseline parathyroid gland volume (mm^3)
Correlation coefficient = 0.25
P = 0.037
Mean intact parathyroid hormone levels (pg/mL)
Baseline parathyroid gland volume (mm^3)
Correlation coefficient = 0.2
P = 0.1
Mean calcium phosphate product levels
Baseline parathyroid gland volume (mm^3)
