# Supplementary material for: Parathyroid gland volume and treatment resistance in patients with secondary hyperparathyroidism: a 4-year retrospective cohort study
Source: Clin Kidney J. 2025 Jan 10;18(2):sfae391. doi: 10.1093/ckj/sfae391 (PMC11803308; doi:10.1093/ckj/sfae391)
Supplement: sfae391_Supplemental_Files [file sfae391_supplemental_files.zip › Supplemental_Tab1.10.29.docx]

**Supplementary Table 1.** Changes in total parathyroid gland volume over 4 years in each subgroup (n = 64)

| Subgroup | Changes in total  parathyroid gland volume (mm^3^) |
| --- | --- |
| All patients | -1.1 (-97.3, 54.2) |
| ≤63 years old | -10.5 (-102.6, 57.1) |
| >64 years old | 1.6 (-60.2, 51.3) |
| Male | -1.1 (-69.9, 58.9) |
| Female | -8.9 (-156.6, 46.6) |
| Dialysis vintage <88 months | 3.9 (-27.2, 66.5) |
| Dialysis vintage ≥88 months | -18.3 (-159.2, 50.8) |
| Intact parathyroid hormone levels <159 pg/mL | 6.3 (-28.3, 66.5) |
| Intact parathyroid hormone levels ≥159 pg/mL | -26.7 (-153.9, 44) |
| Dose of calcimimetics (cinacalcet equivalent) = 0 mg/day | -1 (-41.9, 57.1) |
| Dose of calcimimetics (cinacalcet equivalent) >0 mg/day | -3.1 (-159.2, 51.3) |
| Changes are summarized as median (interquartile range).  This analysis included patients who had undergone two parathyroid gland ultrasonography examinations, with measurable volumes recorded in both. | |
