# Supplementary figures and images for: Parathyroid gland volume and treatment resistance in patients with secondary hyperparathyroidism: a 4-year retrospective cohort study
Source: Clin Kidney J. 2025 Jan 10;18(2):sfae391. doi: 10.1093/ckj/sfae391 (PMC11803308; doi:10.1093/ckj/sfae391)

Subgroup with dialysis vintage < 88 months

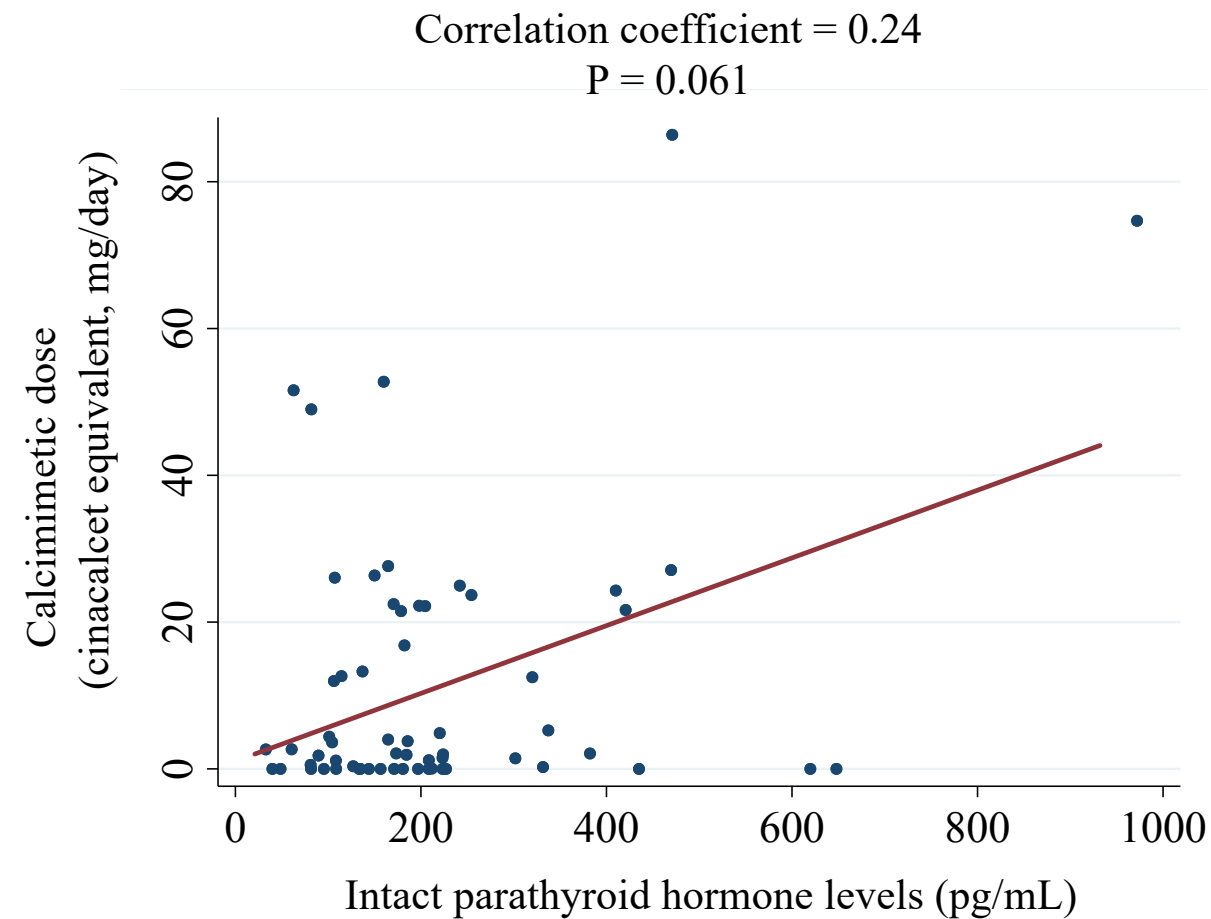

Subgroup with dialysis vintage  $\geq$  88 months

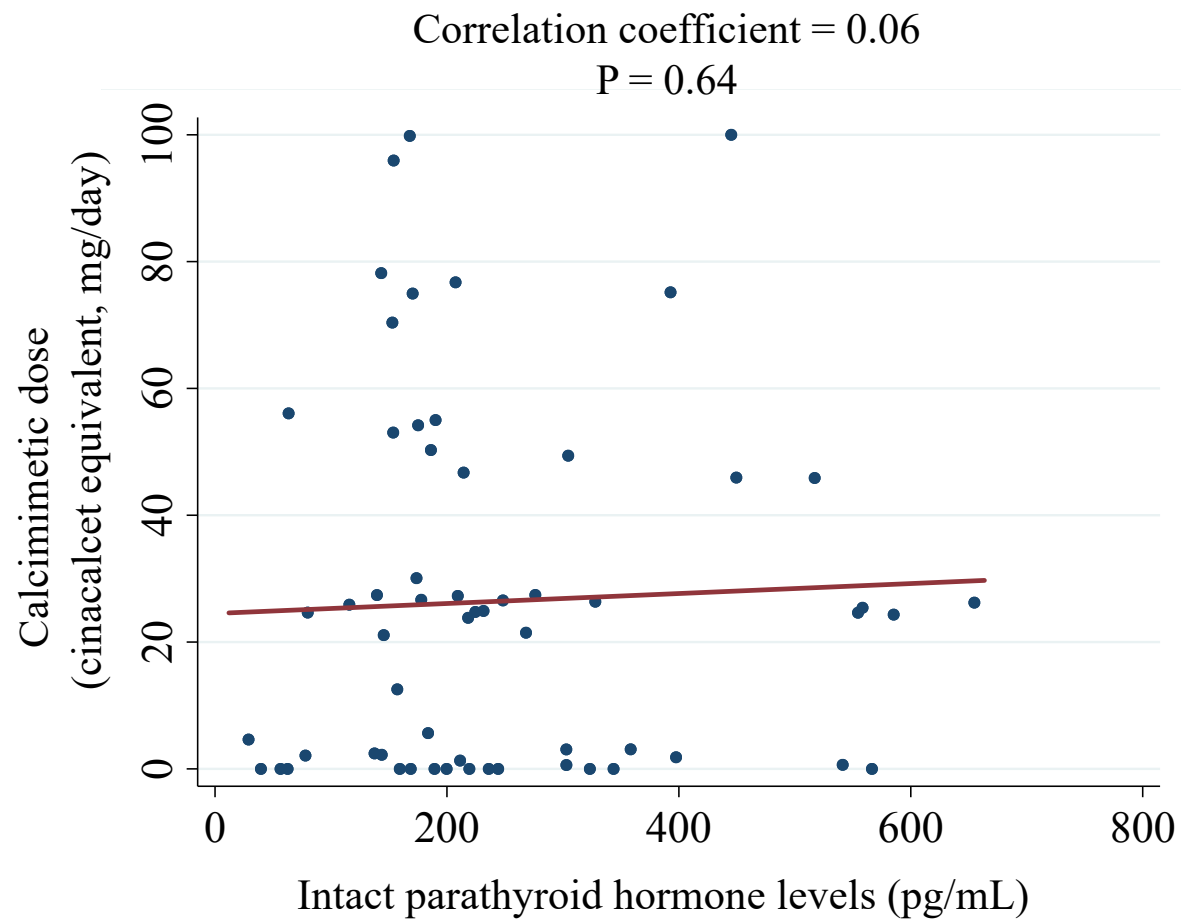

Supplement: sfae391_Supplemental_Files [file sfae391_supplemental_files.zip › Supplemental Figure 3.pdf]

Subgroup with dialysis vintage < 88 months

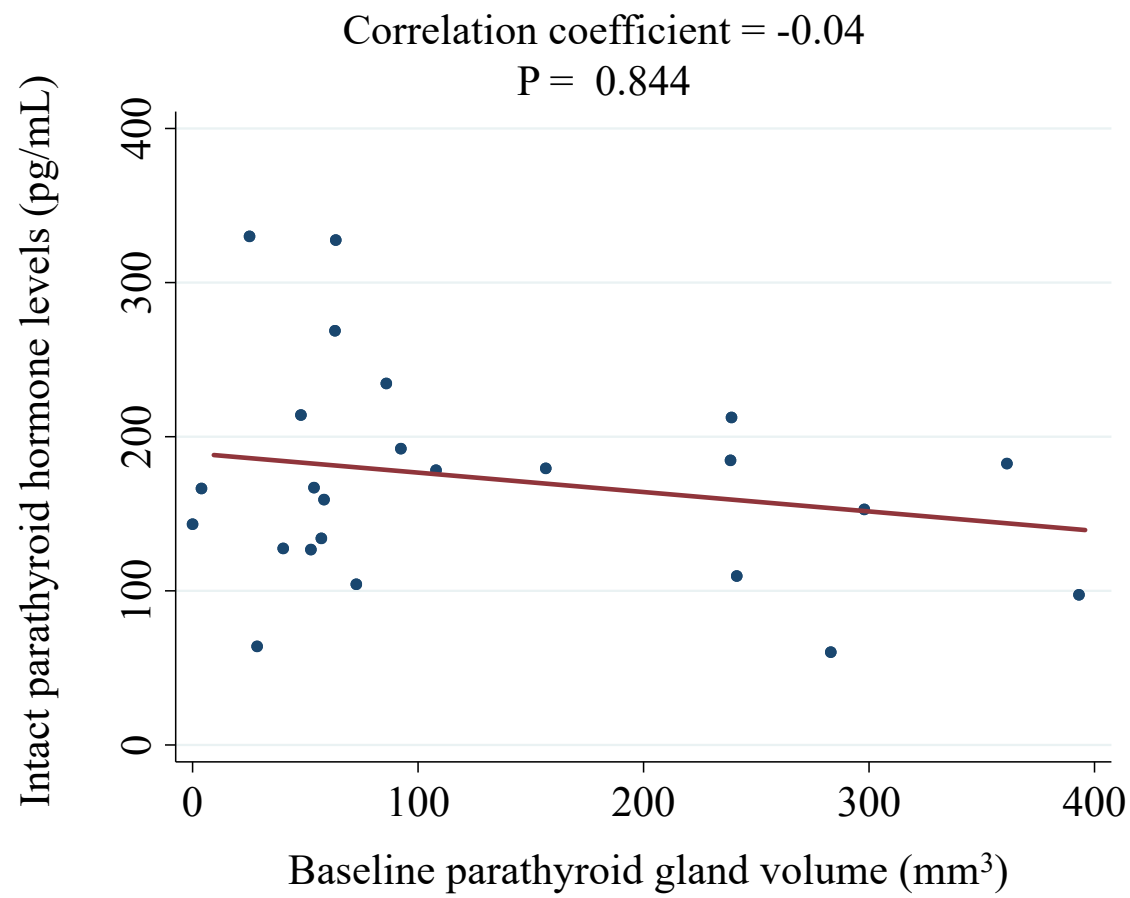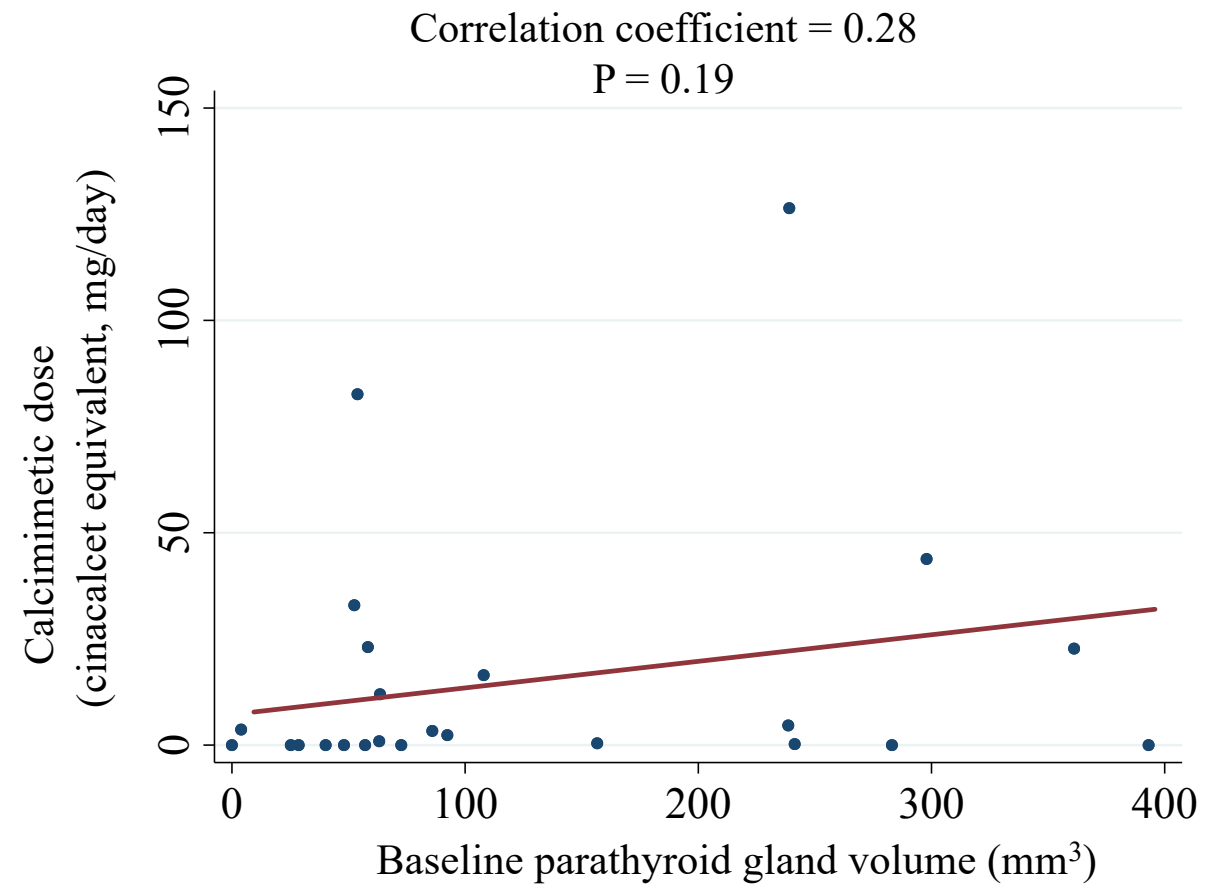

Subgroup with dialysis vintage ≥ 88 months

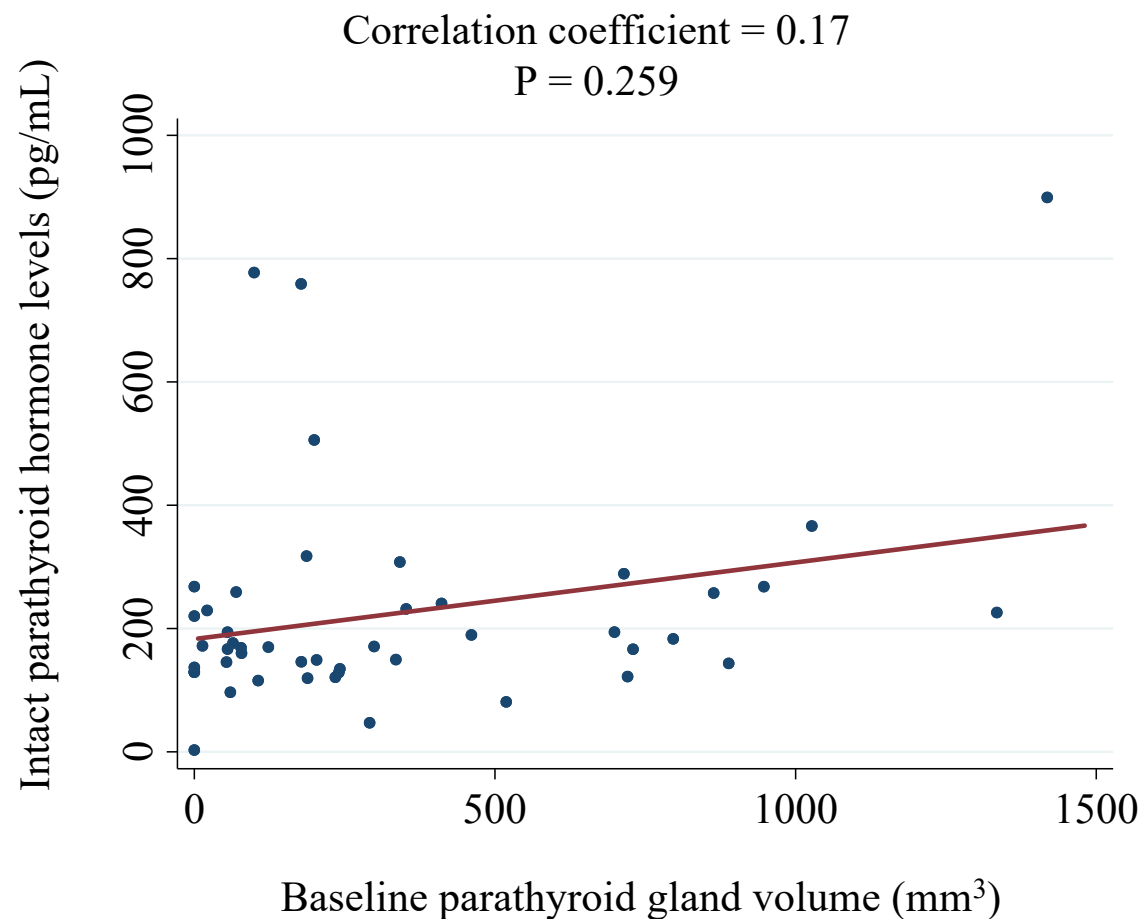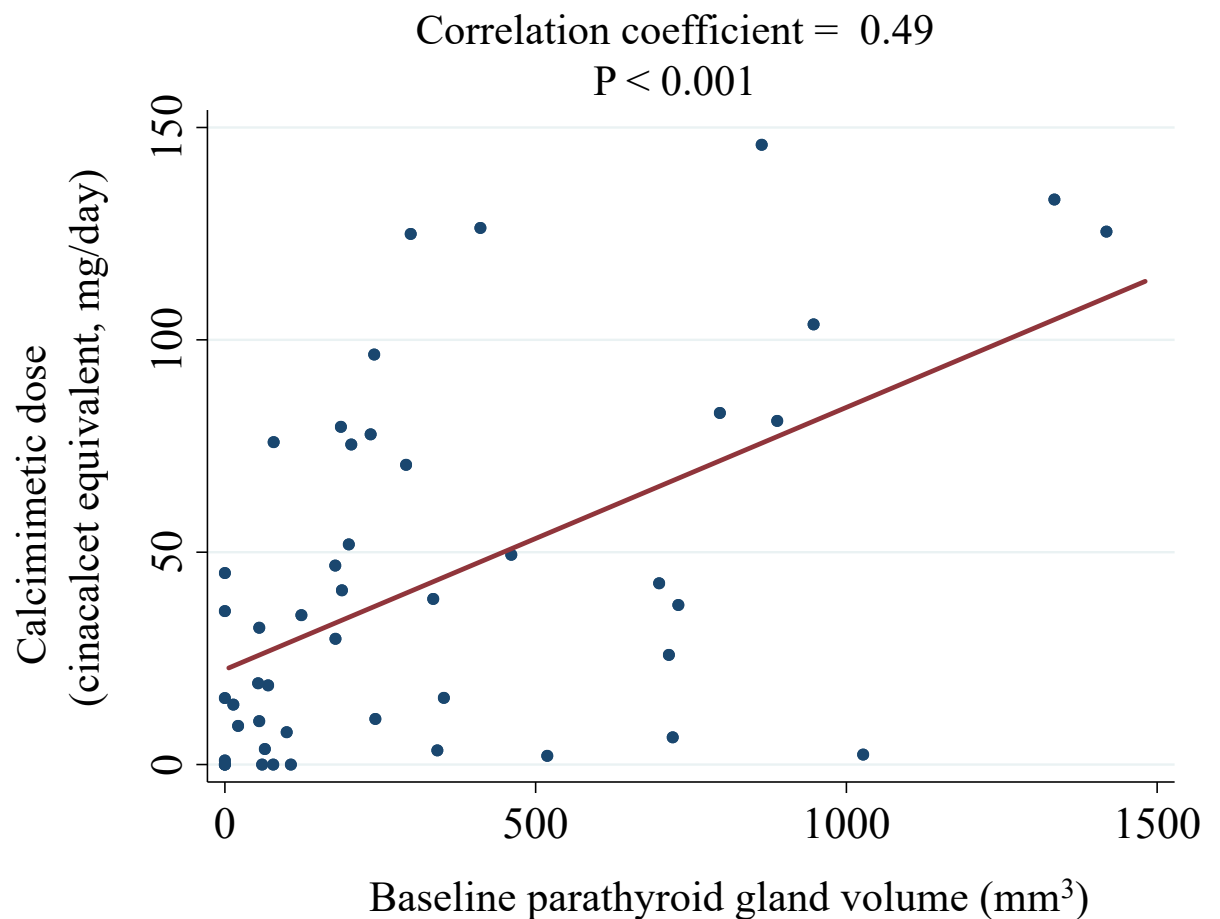

Supplement: sfae391_Supplemental_Files [file sfae391_supplemental_files.zip › Supplemental Figure 4.pdf]
